# Long-term outcomes of single stenting compared with double stenting strategy for unprotected left main coronary artery disease: A protocol for systematic review and meta-analysis

**DOI:** 10.1097/MD.0000000000023639

**Published:** 2020-12-24

**Authors:** Jia-jie Wang, Xin Li, Dong-dong Yan, Zheng Zhang

**Affiliations:** a The first clinical medical college, Lanzhou University; b Department of Cardiology, the first hospital of Lanzhou University, Lanzhou, China.

**Keywords:** Unprotected left main coronary artery disease, single and double stenting strategy, drug-eluting stent, long-term clinical outcomes

## Abstract

**Background:**

The optimal interventions for unprotected left main coronary artery (ULMCA) disease have long been debated, and long-term clinical studies comparing single stenting to double stenting strategies for ULMCA are currently lacking.

**Methods:**

We plan to perform a systematic review and meta-analysis of clinical trials comparing single stenting with double stents strategy for ULMCA disease. We will search PubMed, EMBASE, Web of science and Cochrane Library using a comprehensive strategy. The related conference proceedings and reference lists of the included studies will also be checked to identify additional studies. Two reviewers will screen retrieved records, extract information and assess the risk of bias independently. STATA software will be used to conduct data synthesis. There is no requirement of ethical approval and informed consent.

**Results:**

This study will be submitted to a peer-reviewed journal for publication.

**Conclusion:**

We hope it will provide a relatively comprehensive reference for clinical practice and future relevant clinical trials.

**Ethics and dissemination:**

Ethics approval and patient consent are not required, as this study is a systematic review and meta-analysis.

**INPLASY registration number:**

INPLASY2020110030

## 1 Introduction

Significant left main coronary artery disease occurs in approximately 4% to 6% of patients undergoing coronary angiography.^[1]^ Unprotected left main coronary artery (ULMCA) disease is defined as left main coronary artery disease without right to left collateral circulation or a bridging blood supply.^[2]^ ULMCA is dangerous and presents problem for clinical interventions, meaning the choice of treatment methods is of critical importance. Coronary artery bypass grafting is the recommended gold standard for ULMCA disease.^[3]^ However, with the development of percutaneous coronary intervention techniques and the emergence of drug-eluting stents (DES), percutaneous coronary intervention has emerged as a minimally invasive and therapeutic approach that is comparable to coronary artery bypass grafting intervention.^[4,5]^

Although the single stent (SS) approach of implanting 1 stent in the main branch is the default intervention strategy for ULMCA lesions, double stents (DS) in which stents are implanted in both the main branch and side branch are employed in patients with severely diseased side branches.^[6]^ High quality meta-analyses has been increasingly regarded as 1 of the key tools for achieving evidence of clinical effectiveness.^[7,8]^ However, only a single meta-analysis ^[9]^ published in 2014 compared the outcomes of SS vs DS for ULMCA disease, using odds ratios for their analysis, with no consideration of the effects of time on the outcome. Recent evidence ^[10,11]^ has been published that evaluates the reliability of SS vs DS.

Given the paucity of long-term data assessing either strategy, we performed a systematic review and meta-analysis to assessing the long-term effects of SS vs DS interventions for ULMCA disease in the DES era.

## 2 Methods

### 2.1 Protocol registration

T This protocol was registered with the International Platform of Registered Systematic Review and Meta-Analysis Protocols (INPLASY). The registration number is INPLASY2020110030 (https://inplasy.com//). The content of this protocol will follow the preferred reporting items for systematic review and meta-analysis protocols (PRISMA-P) recommendations. We also plan to conduct it in accordance with the Cochrane Handbook for the Systematic Reviews of Interventions and Preferred Reporting Items for Systematic Review and Meta-Analysis (PRISMA) guidelines.^[12]^

### 2.2 Eligibility criteria

#### 2.2.1 Types of studies

Clinical trials comparing the SS vs DS strategy for ULMCA disease regardless of the specific stenting technique and side-branch size and lesion complexity.

#### 2.2.2 Types of participants

Patients with ULMCA stenoses treated with second-generation DES.

#### 2.2.3 Types of interventions and comparators

The treatment group will be treated with SS strategy, which is implanting 1 stent in the main branch only. The control group will be treated with DS strategy, which is implanting stents in both the main branch and side branch.

#### 2.2.4 Types of outcome measures

The primary outcomes of interest were major adverse cardiovascular events. Secondary outcomes included all-cause mortality, cardiac mortality, target lesion revascularization, myocardial infarction and stent thrombosis (ST). ST was defined according to the academic research consortium definition.^[13]^ Major adverse cardiovascular events was defined as the included trials as the composite of death, target lesion revascularization, myocardial infarction, and ST. Besides, all the endpoints reported in the included studies will be collected and evaluated, although we may not mention some of them in this protocol.

### 2.3 Literature search

A systematic search of online databases including PubMed, EMBASE, Web of science and Cochrane Library will be performed until the end of September 2020 using the keywords “unprotected left main coronary artery disease,” “double stenting strategy” and “drug-eluting stent.” In addition, congress and conference proceedings will be manually retrieved. Related articles and references of included research will also be tracked to find potential studies. If significant data was incomplete in included study, we will contact the authors to get unpublished data.

### 2.4 Study selection and data extraction

After imported into the Endnote X7 and duplication, retrieved records will be independently screened by 2 reviewers (JJW and XL). Firstly, we will read the titles and abstracts of all identified records to exclude clearly unrelated records based on the inclusion criteria. Then the full texts of the articles retained were reviewed to further determine their suitability. Any disagreement will be resolved by a third reviewer (ZZ). We will show the selection process in details in the PRISMA flow chart.^[14]^

Two authors (JJW and XL) of this review will independently extract the data using a pre-defined form. The basic characteristics, related outcome and quality evaluation information of included studies will be collected. Similarly, any discrepancies will be resolved by a third reviewer (ZZ). Data extracted will include author, year, study type, number of participants, intervention, control, population size, patient demographics, procedures, stent type, DS techniques, and outcomes.

### 2.5 Quality of evidence assessment

The quality of included studies will be assessed by Grading of Recommendations Assessment Development and Evaluation (GRADE), and divided into 4 levels: high quality, moderate quality, low quality, and very low quality.^[15]^

### 2.6 Assessment of study bias

Included study bias will be independently assessed by 2 reviewers (JJW and DDY) and any disagreement will be solved by a third reviewer (ZZ). For randomized controlled trials, we will use the Cochrane risk of bias tools to evaluate potential bias in 7 specific domains:

(1) sequence generation,
(2) allocation concealment,
(3) blinding of participants and personnel,
(4) blinding of outcome assessment,
(5) incomplete outcome data,
(6) selective outcome reporting,
(7) other bias.^[16]^

For propensity-match cohort studies, 9-star Newcastle-Ottawa Scale will be applied, which rates studies based on 8 criteria in 3 sources of bias.^[17]^

### 2.7 Statistical Analysis

For dichotomous variables, the Hazard Ratios or Odds Ratios with 95% confidence intervals were calculated from each study. Continuous variables will be presented as standard mean difference with 95% confidence intervals. All endpoints will be combined and performed meta-analysis by using DerSimonian and Laird random effects model.^[18]^ We assessed statistical heterogeneity by using Chi^2^ test and *I*^2^ statistic. We will consider significant heterogeneity when *P* < .10 for Chi^2^ or *I*^2^ > 50%.^[19]^ All primary analyses were performed with STATA v15.1 (Stata Corp, College Station, TX).

#### 2.7.1 Subgroup analysis

We will also conduct subgroup analysis to find more potential information based on pre-set criteria in different follow-up time.

#### 2.7.2 Sensitivity analysis

If the heterogeneity is high, we will conduct sensitivity analyses based on the follow-up time.

#### 2.7.3 Publication bias

The likelihood of publication bias was assessed graphically through the generation of funnel plots, evaluated using an Egger test.^[20]^

## 3 Results

The study does not require ethical approval because the meta-analysis are based on published research and the original data are anonymous. And this study will eventually be published in a peer-reviewed journal in the form of a scientific paper.

## 4 Discussion

Previous cohort studies have not reached consistent conclusions regarding the clinical outcomes of SS vs DS, and follow-up times varied widely among previous cohort studies. The results from our research may provide meaningful evidence for clinical practice and give a valuable reference for future study.

There seem to be some potential limitations for our study. Firstly, we only include English language articles, which might miss some important data in other language article. In addition, according to the initial search result, less random controlled trials and more cohort studies will be included in our study, which may have an obstacle to our data pooling and results interpretation. But it probably helps to promotes several more reliable conclusions and focus on more precious direction for future clinical studies to some extent.

## Author contributions

JJW and ZZ conceived the idea for this study; JJW and XL designed the meta-analysis; JJW and DDY provided statistical advice and input; JJW and XL drafted the protocol; JJW and DDY reviewed the protocol and provided critical feedback.

**Data curation:** Jia-jie Wang.

**Investigation:** Dong-dong Yan.

**Methodology:** Jia-jie Wang, Xin Li.

**Project administration:** Jia-jie Wang, zheng zhang.

**Software:** Xin Li.

**Supervision:** Dong-dong Yan.

**Writing – original draft:** Jia-jie Wang, Xin Li.

**Writing – review & editing:** Dong-dong Yan.
